# Decompressive Hemicraniectomy and Favorable Outcome in a Pediatric Patient with Malignant Middle Cerebral Artery Infarction

**DOI:** 10.1155/2022/6500488

**Published:** 2022-09-13

**Authors:** Ricardo Barrientos, Carlos Sisniega, Samanta Catueno, Robin Hougen, Ashley Hanna, Utpal Bhalala

**Affiliations:** Driscoll Children's Hospital, Corpus Christi, TX, USA

## Abstract

We report a rare case of middle cerebral artery (MCA) stroke in a teenage girl with initial improvement, followed by progression to malignant MCA infarction, requiring an urgent decompressive hemicraniectomy (DHC). Additionally, we report improvement in all areas, including language, comprehension, and motor skills at discharge and the 4-month follow-up. This rare presentation highlights the importance of monitoring the neurological status of a patient with an MCA infarct for progression to a life-threatening malignant MCA infarct. This case report also highlights the importance of consideration of DHC for a favorable outcome of the MMCA infarction.

## 1. Introduction

Large ischemic strokes presenting as malignant cerebral edema account for 1%–10% of all ischemic strokes [1]. Occlusion of the middle cerebral artery (MCA) leads to significant cerebral ischemic infarction. Hypodensity of more than 50–75% of the MCA territory, including the basal ganglia, involvement of additional vascular territories, and a cerebral midline shift of more than 4 mm at the level of the pineal gland in the initial 48 h indicate life-threatening infarct volume, i.e., malignant cerebral infarction [2, 3]. Neurological deterioration occurs within 5 days, with the highest frequency of deaths due to transtentorial herniation and subsequent brain death on day 3 [4]. Mortality rates have been reported to be as high as 80%, and most of the survivors are left severely disabled [5, 6]. The goal of surgical intervention is to save lives, minimize further neurologic deterioration, and achieve the best neurological outcome for the patient [7].

We hereby report a teenage girl who presented to our tertiary care children's hospital with a rare malignant middle cerebral artery stroke, with a brief honeymoon period before clinical worsening, who had a favorable outcome after an emergency decompressive hemicraniectomy (DHC). This case is important to report in the literature because of the rarity of MMCA in children, especially with delayed worsening, and the ultimately favorable short-term and long-term outcomes following emergency DHC.

## 2. Case History

**Figure 1:**
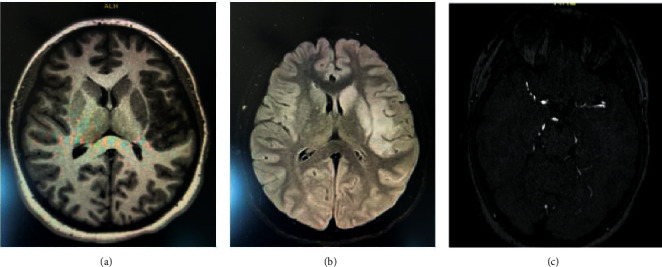
(a) : MRI brain WO contrast. An MRI of the brain showed an acute infarct without hemorrhage involving the left insula and territory of the left middle cerebral artery. (b) MRI brain W-WO contrast. There is a loss of appropriate signal intensity within the M1 segment of the left MCA for a length of appropriate 9 mm. There is a reconstitution of appropriate signal intensity distal to the aforementioned. There is diminutive signal intensity involving the supraclinoid aspect of the right ICA with appropriate reconstitution distally, which may be artifactual in nature as no restricted diffusion is seen within the right cerebral hemisphere. The parent branches, the ACAs, PCAs, basilar artery, and vertebral arteries appear without significant stenosis. (c) MRI angiogram with head WO contrast. There is a demonstration of restricted diffusion involving the left basal ganglia and portions of the left frontal, parietal, and temporal lobes.

A 14-year-old previously healthy female presented to an outside emergency room (ER) with loss of consciousness for 45 minutes followed by right side weakness without seizure-like activity or vomiting. Her initial CT scan and CTA of the head and neck were reported to be negative. MRI of the brain showed an acute infarct without hemorrhage involving the left insula, a territory of the left middle cerebral artery (Figure 1)(a), requiring subsequent transfer to our institution's pediatric intensive care unit (PICU) for further management.

Upon arrival at our institution, the patient was hemodynamically stable, afebrile, and awake. She appeared alert, moaned in response to questions, and followed some commands. By the time she arrived at our PICU, she was beyond the timeline/window for receiving systemic, thrombolysis and therefore we managed her per our stroke protocol and antiplatelet therapy. The initial diagnostic workup for the etiology of her underlying stroke was suggestive of idiopathic MMCA (Table 1).

Given her symptomatic infarct and family history of multiple miscarriages, the patient was placed on subcutaneous low molecular weight heparin with the goal of reaching therapeutic anticoagulation. She had right-sided hemiplegia with an absent light touch on the right, and the National Institute of Health Stroke Scale (NIHSS) score was 19. The findings of the MRI and MR angiogram are shown in Figure 1.

**Figure 2:**
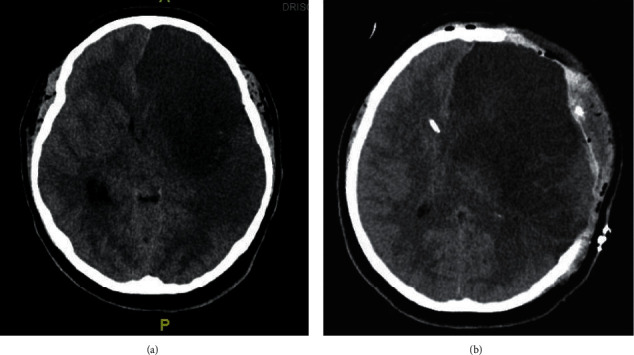
(a) : CT scan head WO contrast. A large hypodensity of the left MCA territory, predominantly involving the left frontal lobe, basal ganglia, insula, and left temporoparietal lobes; worsened since the prior MRI study. There is a significant mass effect on the left lateral ventricle. There is an interval development of uncal herniation and anterior subfalcine measuring 7-8 mm towards the right. There is an obliteration of the suprasellar and perimesencephalic cisterns. (b) CT Scan head WO contrast.

On hospital day 3, the patient remained hemodynamically stable and showed neurologic improvement with improved consciousness. Her NIHSS improved from 19 on admission to 15, and she was transferred to the inpatient ward. On the evening of hospital day 5, she had progressively worsening headaches and emesis, with regression in neurologic function, and was ultimately found to be unresponsive with unequal pupils. The CT scan of the head without contrast demonstrated significant progression of her known MCA infarct, with malignant cerebral edema, midline shift, and effacement of the left lateral ventricle. There was interval development of uncal and subfalcine herniation (Figure 2(a)). The patient was intubated and taken emergently to the operating room (OR) for a left-sided DHC with duraplasty and insertion of an external ventricular drain (EVD). Following her DHC, she was managed in the PICU with neuroprotective strategies, aggressive management of her elevated intracranial pressure, and goal-directed management of her cerebral perfusion pressure.

**Figure 3:**
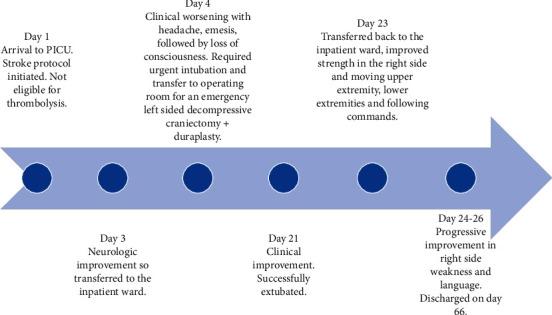
Timeline of patient course throughout hospitalization.

On hospital day 13, a follow-up CT scan of the brain without contrast showed interval improvement of edema, midline shift, and mass effect on the lateral and third ventricles (Figure 2)(b). On hospital day 20, after an appropriate weaning trial, the EVD was removed. On hospital day 21, she was successfully extubated with a high-flow nasal cannula. On hospital day 23, the patient was transferred back to the inpatient ward, hemodynamically stable, with improved strength and spontaneous movements in the right upper and lower extremities. On hospital day 66, the patient continued to show steady improvement in physical and occupational therapies and speech therapy and was discharged in a stable condition to the inpatient rehabilitation center (Figure 3).

At a 4-month follow-up in the neurosurgery clinic, she was alert, interactive, verbal with slow speech, able to identify simple objects, move her left upper and lower extremities well, lift her right shoulder, and extend the right lower extremity.

## 3. Discussion

Our case described above is a unique report of DHC in a pediatric patient who had a rare malignant middle cerebral artery infarction with a positive outcome. In 2013, Shah et al. described 3 cases of MMCA in children who were successfully managed with DHC, and in the same report, they also reviewed 26 similar cases described in 12 prior case series [8]. Overall, there is a paucity of literature on pediatric MMCA, and none of the prior reports described delayed progression warranting urgent surgical treatment and aggressive medical management. Our case of pediatric MMCA was unique with a brief honeymoon period of neurologic improvement (NIHSS score improving from 19 to 15) in the first 72 hours of presentation before the stroke turned malignant. Since cerebrovascular catastrophe manifested as ischemic and/or hemorrhagic strokes is far more common in adults as compared to children, there is ample literature on MMCA and DHC for MMCA in adults [9–11]. Pooled analysis of the European trials provides Class I evidence for the performance of early DHC in the setting of large unilateral infarcts within 48 hours of the ischemic event [12]. Given the potential tradeoff between survival and a good neurological outcome, frank discussion with the patient and the family should be held early in the setting of an envisioned need for surgical decompression, keeping in mind that two randomized control trials reported a trend of reduced disability among survivors [13].

A retrospective study of MMCA among 10 children from 5 different children's hospitals showed that all 3 children who did not undergo DHC developed intractable intracranial hypertension and ultimately fulfilled the criteria for brain death, whereas the remaining 7 who underwent DHC survived, with rapid improvement in their level of consciousness postoperatively [14]. Similar to these cases of DHC, our case of MMCA reports favorable outcomes following DHC. Unlike our case, none of the previous pediatric cases of MMCA described any brief period of neurologic improvement before the clinical deterioration requiring urgent DHC. This is one of the unique features in this case that is worth reporting. Due to neuroplasticity, children are likely to benefit from more aggressive therapies, such as DHC [15], and therefore surgical decompression should be considered in MMCA in children.

Postsurgical changes include left frontoparietal and temporal craniectomy, scalp edema with a small amount of hemorrhage, and air pockets. Surgical drains terminate in the left temporal scalp. Interval placement of a right frontal approach shunt catheter terminating in the body of the right lateral ventricle.

## 4. Conclusions

The use of DHC in children with malignant middle cerebral artery infarction has not been extensively evaluated. We report a pediatric case of MCA stroke with initial improvement, followed by progression to MMCA requiring an urgent DHC. Additionally, we report short-term and long-term improvements following the DHC.

## Figures and Tables

**Table 1:** Initial diagnostic workup.

Study	Results
CBC	WBC 15.7 cells/L, HB 10.9 gm/dL
	HCT 34.1 L/L, PLT 362 per microL
CMP	Na+ 140 mEq/L, K+ 3.6 mEq/L, Ca 8.7 mg/dL
	BUN 6 mg/dL, creatinine 0.42 mg/dL
PT	13.9 seconds
INR	1
APTT	27 seconds
Fibrinogen	357 mg/dL
Lipid panel	LDL 117 mg/dL/HDL 31 mg/dL
POCT glucose	98 mmol/L
MTHFR, DNA^*∗*^	C667T- detected heterozygous
	A1298 C—not detected
Homocysteine total	5.4 *µ*mol/L
Lactate level	0.48 mmol/L
Protein C	107 *µ*g/ml
Protein S functional	95%
Lupus anticoagulation panel	Not detected
Antiphospholipid antibody panel	Negative
Factor leiden	Negative
Anti-ds DNA antibody	Negative
Sjogren's abs	Negative
Sickle cell screen	Negative
APC resistance	2.5
ATIII^*∗∗*^	80%
Factor II, DNA	Negative
Antixa assay	0.7 units/mL
Factor 8^∗∗∗^	214%
COVID-19 IgG/IgM antibody	Negative
Urine drug screen^∗∗∗∗^	Benzodiazepine

CBC= Complete blood count. CMP= Complete metabolic profile. PT= Prothrombin time. INR= International normalized ratio. APTT = Activated partial prothrombin time. POCT= Point of care testing. MTHFR, DNA = Methylenetetrahydrofolate reductase. Anti-Ds DNA = Anti-double stranded DNA. APC = Activated protein C. ATIII = Antithrombin III. AntiXa = Anti-factor Xa. ^*∗*^This result is not associated with an increased risk for hyperhomocysteinemia. ^*∗∗*^Low-normal, not clinically relevant. ^∗∗∗^Thrombophilia workup negative. ^∗∗∗∗^Received intravenous benzodiazepine for suspected seizure/agitation on initial presentation.

## Data Availability

The data used to support the findings of this study are available from the author upon request.
